# Association of Serum Glucose, Serotonin, Aspartate Aminotransferase, and Calcium Levels with Meat Quality and Palatability Characteristics of Broiler *Pectoralis Major* Muscle

**DOI:** 10.3390/ani12121567

**Published:** 2022-06-17

**Authors:** Boin Lee, Young Min Choi

**Affiliations:** Department of Animal Sciences and Biotechnology, Kyungpook National University, Sangju-si 37224, Korea; ananassab@knu.ac.kr

**Keywords:** serum biochemical parameters, apoptosis, meat quality, palatability, chicken breast

## Abstract

**Simple Summary:**

Apoptosis is a critical and highly regulated cell death process after exsanguination, and serum apoptosis-related molecules are associated with apoptotic and glycolytic potentials. Thus, the activities and production of these molecules can directly and/or indirectly affect variation in meat quality characteristics. However, there is limited information on the complex relationship between serum biochemical molecules and their effects on the meat and eating quality of broilers. In the present study, the activity of aspartate aminotransferase, a cytoprotective molecule, was found to be associated with other apoptosis-related molecules, including blood glucose, serotonin, and calcium levels, at exsanguination. All serum parameters analyzed in this study were related to the early postmortem and ultimate muscle pH. Thus, apoptosis-related molecules may affect the glycolytic rate in broiler *pectoralis major* muscle. Additionally, our results demonstrated that the variations in the sensory quality traits of broilers could be somewhat explained by the levels of apoptosis-related serum parameters at exsanguination.

**Abstract:**

This study investigated the correlations between apoptosis-related blood biochemical parameters measured at exsanguination and the meat and sensory quality characteristics of broiler pectoralis major muscle. The concentration of serotonin showed a positive correlation with aspartate aminotransferase (AST) activity (*p* < 0.001) and a negative correlation with calcium content (*p* < 0.01). All serum parameters showed relationships with muscle pH at 15 min and/or 24 h postmortem (*p* < 0.05). Serum AST activity, which had a negative correlation with calcium content (*p* < 0.01), was positively related with muscle pH and negatively correlated with Warner–Bratzler shear force values (WBS, *p* < 0.05). Principal component analysis results revealed the associations between AST activity and meat quality traits, including pH_24h_, lightness, and WBS. Furthermore, cooked breast with higher AST activity and lower calcium level tended to exhibit higher scores of tenderness and overall acceptability than that with lower AST activity and higher calcium level (*p* < 0.05).

## 1. Introduction

Poultry meat production has been rapidly increasing worldwide with changing food consumption trends [1]. Given the consumer trend, visual appearance and technical quality of each chicken part are becoming more important aspects of consumer decisions [2,3]. Thus, the poultry industry has been aiming to improve meat quality and reduce the incidence of muscular abnormalities, such as pale, soft, and exudative (PSE)-like conditions, in chicken *pectoralis major* (PM) muscle [4,5]. It is well-known that meat quality characteristics are influenced by genetic and environmental factors [6], and their deterioration is the result of complex endocrinological, physiological, and metabolic changes in the skeletal muscle during the postmortem period [7,8]. Thus, before and after exsanguination, the levels of produced and secreted substances via bio-physicochemical changes, directly and/or indirectly affect the meat and organoleptic quality characteristics [8].

Under ischemic conditions, serotonin—a monoamine neurotransmitter—plays a cytoprotective role in threatening situations such as apoptosis to maintain the normal physiological equilibrium [9,10]. Additionally, serum aspartate aminotransferase (AST; also known as glutamic-oxaloacetic transaminase) and cytoplasmic free calcium are also associated with apoptosis and glycolysis [11,12]. In the postmortem muscle, lower AST levels can lead to an increase in the level of cytoplasmic free calcium level via the increased depolarization of the mitochondrion. This phenomenon, in turn, increases the activity of calcium-mediated proteases [12]. In contrast, AST regulates glucose level by inhibiting the expression of glycolytic enzymes to maintain normal glucose homeostasis [11]. Thus, these serum biochemical substances could be useful indicators of stress susceptibility, cell damage by apoptosis, and/or meat quality characteristics [8,13,14]. However, there is only limited information on the complex relationship between blood biochemical substances and their effects on the meat quality in broilers. Therefore, this study investigated the relationships between the blood biochemical parameters associated with glycolysis and cell apoptosis, including blood glucose, serotonin, AST, and calcium, with meat quality and palatability characteristics in chicken PM muscle.

## 2. Materials and Methods

### 2.1. Animals and Muscle Samples

One hundred and five male Ross 308 broilers (4 wk of age; average live weight 1374 ± 109 g) were used in this study. All birds were reared on a commercial farm and fed the same commercial diet in accordance with the National Research Council. The environmental conditions for birds were the same before and after slaughter. Chicks were slaughtered at a commercial abattoir (Gyeongsanbuk-do, Korea) in four batches (25 to 30 birds per batch) under the supervision of the Korean grading service for animal products. After bleeding, blood glucose level was immediately measured using a glucose monitoring device (ACCU CHEK, Roche Diagnostics GmbH, Mannheim, Germany). PM muscle pH was measured at 15 min postmortem. At 24 h postmortem, a total of 105 left PM muscles were used to assess meat quality characteristics, including pH_24h_, meat color, drip loss, cooking loss, and Warner–Bratzler shear force (WBS); the remaining right PM muscles were stored at −20 °C for later evaluation of sensory quality characteristics.

### 2.2. Blood Parmeter Measurements

A blood sample was collected from the left jugular vein of each bird, placed in a serum separating tube (BD Vacutainer, Plymouth, UK), and coated with clotting agent to separate the serum, which was immediately gently shaken. Collected blood samples were centrifuged at 1000× *g* for 15 min at 4 °C, and the supernatant plasma samples were stored at −20 °C until the assessment of serotonin, AST, and free calcium. Serotonin level was measured using enzyme-linked immunosorbent assay (ELISA) kits (ADI-900-175, Enzo Life Science, Farmingdale, NY, USA) following the manufacturer’s instructions. According to Reitman and Frankel [15], AST activity was analyzed using an automatic analyzer (Flexor EL 200, Elitech, Paris, France) with a commercial kit (Elical, Elitech). Free calcium concentration was determined using a colorimetric assay kit (QuantiChrom, BioAssay Systems, Hayward, CA, USA) according to the instructions provided by the manufacturer.

### 2.3. Meat Quality Characteristics

A pH instrument equipped with a penetration probe (Testo 206-pH2, Testo Inc., Lenzkirch, Germany) was used to measure the pH at the surface of the left PM muscle at 15 min and 24 h postmortem. At 24 h postmortem, the surface color was assessed from the upper part of the breast muscle using a Minolta chromameter (CR-400, Minolta Camera Co., Osaka, Japan) in a cold room (4 °C). Lightness (L*), redness (a*), and yellowness (b*) were expressed in accordance with the recommendations of the Commission Internationale de l’Eclairage [16]. After storing the muscle samples at 4 °C for 48 h, drip loss was calculated as the percentage of the difference in the sample weight before and after storage [17]. Cooking loss and WBS value were evaluated based on the methods outlined by Honikel [17] and the American Meat Science Association [18], respectively. To assess cooking loss, each weighed sample was put into a closed polyethylene bag, and then placed in a temperature-controlled water bath (80 °C) until the core temperature reached 71 °C. Heated samples were cooled in an ice-slurry until equilibration, then weighed again to calculate the percentage of weight loss. After cooking loss, cooked samples were trimmed into cylindrical shapes (more than six samples, 1.27 cm-diameter, parallel to the direction of the muscle fiber) for WBS analysis, which was performed using an Instron Universal Testing Machine (cross head speed of 200 mm/min; Model 1011, Instron Corp., Canton, MA, USA).

### 2.4. Sensory Quality Evaluation

A total of 105 right PM samples were randomly coded with a three-digit number and evaluated over 21 sessions (5 samples per session). Twelve sensory panelists (six women and six men; aged 24 to 45 years) were selected from the researchers and faculty of Kyungpook National University (KNU) and trained according to the procedures of AMSA [18] and Meilgaard et al. [19]. All training and testing was conducted at KNU, and human ethics approval was granted by the Bioethics Committee of KNU (protocol number: 2019-0027). Before each sensory evaluation session, frozen samples were thawed overnight at 4 °C. Thawed samples were roasted by pan-frying at 180 °C using an electric induction range (CIR-IH300RGL, Cuchen, Seoul, Korea) set to the fifth heating level. During the sensory quality assessment, trained panelists were provided with unsalted crackers and sufficient water prior to ingesting the first sample and between samples to refresh their plate. Sensory quality traits were evaluated for eight attributes (i.e., initial tenderness, rate of breakdown, amount of perceptible residue, overall tenderness, juiciness, flavor intensity, off-flavor intensity, and overall acceptability) using a nine-point scale.

### 2.5. Statistical Analysis

Blood biochemical parameter data were analyzed using the SAS software (SAS Institute, Cary, NC, USA) to calculate simple means, standard deviations, minimums, and maximums. Spearman correlation coefficients were calculated using the PROC CORR procedure in the SAS software to correlate the blood biochemical parameters with the meat and sensory quality characteristics. Principal component analysis (PCA) was performed based on the correlation matrix using the PRINCOMP procedure in SAS software.

## 3. Results

### 3.1. Relationship between Blood Biochemical Parameters

Table 1 presents the mean, standard deviation, and overall range (minimum and maximum) for all measured parameters, including blood glucose, serotonin, AST, and free calcium. Broad ranges were observed for biochemical parameters, especially blood glucose, serotonin, and AST levels. The correlations between blood parameters are shown in Table 2. Blood glucose level was negatively correlated with AST activity (*r* = −0.21, *p* < 0.05), and serotonin concentration was associated with AST activity (*r* = 0.38, *p* < 0.001) and free calcium level (*r* = −0.32, *p* < 0.01). A negative correlation was also observed between AST activity and calcium content (*r* = −0.43, *p* < 0.001). However, no significant correlation was observed between concentrations of free calcium and blood glucose (*p* > 0.05).

### 3.2. Relationship between Blood Biochemical Parameters and Meat Quality Characteristics

The correlations between blood parameters and meat quality traits are presented in Table 3. Muscle pH_15min_ was positively correlated with the levels of serotonin (*r* = 0.36, *p* < 0.001) and AST (*r* = 0.21, *p* < 0.05). All blood biochemical parameters except serotonin level (*p* > 0.05) were related to the ultimate muscle pH (*p* < 0.05). In the meat surface color, there were no correlations between lightness and any serum parameters (*p* > 0.05), whereas redness had a relationship with serotonin (*r* = −0.28, *p* < 0.01), AST (*r* = −0.53, *p* < 0.001), and calcium (*r* = 0.37, *p* < 0.001) levels. In contrast, drip loss did not correlate with any serum parameters (*p* > 0.05). Negative correlations were found between cooking loss and serotonin level (*r* = −0.36, *p* < 0.001) and between WBS value and serum AST activity (*r* = −0.21, *p* < 0.05).

### 3.3. Relationship between Blood Biochemical Parameters and Sensory Quality Characteristics

Table 4 shows Pearson’s correlation coefficients for the relationships between the serum biochemical parameters and palatability characteristics of broiler PM muscles. No sensory quality traits correlated with blood glucose concentration (*p* > 0.05). Off-flavor intensity score was positively correlated with serotonin level (*r* = 0.28, *p* < 0.01), and a negative correlation was observed between the score of the amount of perceptible residue and concentration of free calcium (*r* = −0.20, *p* < 0.05). Meanwhile, significant associations were observed between palatability characteristics and blood AST activity (*p* < 0.05); however, initial tenderness did not correlate with AST level (*p* > 0.05).

### 3.4. PCA Results

**Figure 1:**
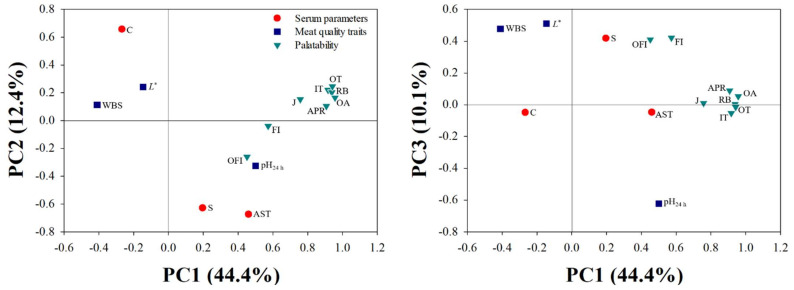
Principal component analysis (PCA) plots for PC1 vs. PC2 and PC1 vs. PC3. The percentage of variance explained by the three PCs was 66.9%. S, serotonin; AST, aspartate aminotransferase; C, calcium; L*, lightness; WBS, Warner–Bratzler shear force; IT, initial tenderness; RB, rate of breakdown; APR, amount of perceptible residue; OT, overall tenderness; J, juiciness; FI, flavor intensity; OFI, off-flavor intensity; OA, overall acceptability.

A PCA was conducted to understand the relationship between the variables via a reduction in the data dimensionality, including blood biochemical parameters, meat quality traits, and sensory quality characteristics (Figure 1). The first three principal components (PCs) explained approximately 67% of the variance observed in the 14 variables; PC1 explained 44.4% variability, whereas PC2 and PC3 explained 12.4% and 10.1% variability, respectively. AST and calcium levels were placed on the opposite quadrants in PC1, indicating their negative correlation. Serotonin level and AST activity were distributed in the area with positive PC2 loading. Blood calcium content, lightness, and WBS were distributed in the area with positive loading of PC1; in contrast, calcium content, muscle pH_24h_, and all sensory quality characteristics were distinguishable by the negative loading of PC1.

## 4. Discussion

After exsanguination, cells undergo programmed cell death, a well-regulated and complex energy-dependent process; thus, the apoptotic mechanism is the first step in the conversion of muscle to meat [20]. Particularly, glucose metabolism can play a key role in the early apoptotic paradigm, as the amount and consumption rate of glucose during early postmortem determines the extent of ATP depletion and mitochondrial death [21]. The extent of apoptotic changes in muscle fibers during the postmortem period is influenced by various factors, such as the animal breed and individual characteristics [4]. Each individual animal tends to exhibit different levels of production and activity of serum biochemical parameters during the early postmortem period, even under the same environmental conditions [22]. These molecules interact with each other and influence the apoptotic process [8]. Choe et al. [23] reported that animals having greater glycolytic potentials exhibit a higher level of blood glucose and faster rate of ATP depletion compared with animals having lower glycolytic potentials. Meanwhile, serotonin, secreted by the serotonergic systems, plays an essential role in the regulation of several neurological functions, such as mood, stress, aggression, feelings, cognition, and behavior [24], and this molecule is involved in glucose homeostasis and lipid metabolism [25]. Thus, blood glucose and serotonin have been suggested as parameters indicating the levels of stress and muscle damage [26]. Although serotonin is responsible for hypoglycemia [27], the correlation between these molecules may not be detected as being affected by a variety of factors. In the current study, broad ranges of blood glucose, serotonin, and AST levels may be associated with differences in the extent of individual stress susceptibility. The blood glucose concentrations measured immediately after exsanguination did not show any association with the concentration of serotonin (*p* > 0.05).

AST is a ubiquitous enzyme found in significant concentrations in the liver, skeletal muscle, cardiac muscle, and brain [28]. Its level of this enzyme could be reflective of the extent of damage and fatigue in the liver or skeletal muscle of living animals, since the level is increased following cellular injury [29]. In damaged cells, AST is essential for cell survival because it contributes to cell proliferation and inhibits mitochondrial disintegration by regulating calcium release into the cytoplasm [12]. This implies that their relationship plays a critical role in the apoptotic process [12]. The relationship between AST and calcium is relevant to the results of this study. In the current study, serum AST activity increased as the free calcium level decreased at exsanguination (*p* < 0.05). On the other hand, AST activity and blood glucose concentration had a negative correlation (*p* < 0.05). The positive correlation between serum AST and serotonin levels of broilers noted in this study (*p* < 0.001) can be explained by the biochemical functions of these two molecules; both these biomolecules protect the cells against a variety of biophysiochemical injuries [12,25]. PCA results revealed similar relationships wherein PC2 displayed a positive association with blood AST and serotonin levels and a negative association with AST and calcium levels.

During the conversion of muscle to meat, glycolysis is one of the key metabolic pathways [30]. Stress-susceptible animals commonly exhibit a higher blood glucose level through the rapid depletion of glycogen that results in increased lactate production compared with stress-resistant animals [30]. In pigs and chickens, both known to be stress-susceptible animals, the muscles showing a greater glycolytic potential may experience a sharp decrease in pH during the early postmortem owing to increased lactate accumulation and are more likely to produce inferior meat quality traits, such as a paler color, lower water holding capacity, and tougher cooked meat, than the muscles showing lower glycolytic potential [31]. Choe et al. [23] suggested that blood glucose at exsanguination can be a useful indicator to explain the variation in pork quality, as it is negatively correlated with muscle pH_45min_ and pH_24h_ and positively correlated with meat surface lightness and drip loss. However, in the broilers, aside from the negative relationship between blood glucose levels at exsanguination and muscle ultimate pH (*p* < 0.05), there were no significant correlations with other meat quality characteristics (*p* > 0.05). Several studies have reported associations between stress-related hormones and meat quality characteristics [8,13,32] because these hormones can regulate the rate and extent of glycolysis via the modulation of glucose consumption [13,27]. However, the levels of stress-related hormones can vary depending on the characteristics of preslaughter stressors (e.g., the type, duration, and intensity of stress) and individual susceptibility to stress [8]. Ferguson and Warner [32] suggested that the effect of serotonin, which attenuates stress response before slaughter, on the meat quality variations is somewhat inconclusive. In the present study, the concentration of serotonin had a limited effect on meat and sensory quality characteristics (*p* > 0.05), although it was associated with muscle pH_15min_, cooking loss, and off-flavor intensity (*p* < 0.05).

Considering the association between AST and calcium, increased AST activity can inhibit the release of apoptotic factors as well as inhibit the activation of calcium-mediated enzymes through to maintain calcium homeostasis during the postmortem period, whereas a lower activity of AST may be accompanied by a rapid apoptosis process [12]. Guo et al. [33] reported that an increased in the apoptotic potential caused by a calcium channel disorder results in muscular damage and deterioration of meat quality and is also associated with the occurrence of PSE meat. In the current study, owing to the cytoprotective role of AST, broilers with a higher serum AST activity exhibited a slower pH decline and lower WBS values, which resulted in less force required to break down the cooked meat with more moisture in the mouth after chewing compared with broilers with a lower serum AST activity (*p* < 0.05). However, correlation analysis may not accurately describe cause–effect interactions between variables. Thus, a PCA was performed, showing that blood calcium content, which was negatively correlated with AST activity and ultimate pH, was located close to the lightness and WBS values. Additionally, serum calcium and all palatability, except for flavor and off-flavor intensities, were distributed in the area with negative loading of PC1. Thus, the combination of low AST activity and high calcium level may lead to a faster pH decline and produce inferior quality meat in the PM muscles of broilers.

## 5. Conclusions

The results of this study demonstrate that serum AST activity significantly correlated with the levels of serum glucose, serotonin, and calcium measured at exsanguination, and all blood parameters were associated with the glycolytic rate measured by muscle pH. Particularly, the levels and combinations of serum molecules, including AST activity and calcium content, can be useful indicators to explain variations in the meat quality and palatability traits of broiler breasts.

## Figures and Tables

**Table 1:** Serum biochemical parameters measured at exsanguination of broilers.

	Mean	Standard Deviation	Minimum	Maximum
Blood glucose (mg/dL)	260	42.4	189	460
Serotonin (ng/mL)	1035	520	98.6	2584
AST (U/L)	239	40.3	179	337
Calcium (mg/dL)	11.0	0.9	9.00	13.9

Abbreviation: AST, aspartate aminotransferase.

**Table 2:** Correlation coefficients between the serum biochemical parameters of broilers.

	Serotonin	AST	Calcium
Blood glucose	−0.04	−0.21 *	0.10
Serotonin		0.38 ***	−0.32 **
AST			−0.43 **

Levels of significance: * *p* < 0.05; ** *p* < 0.01; *** *p* < 0.001. Abbreviation: AST, aspartate aminotransferase.

**Table 3:** Correlation coefficients between the serum biochemical parameters and meat quality traits of broilers.

	pH_15min_	pH_24h_	L*	a*	b*	Drip Loss	CL	WBS
Blood glucose	0.05	−0.25 **	0.09	0.08	−0.02	0.07	−0.11	−0.01
Serotonin	0.36 ***	0.05	−0.17	−0.28 **	0.15	0.09	−0.36 ***	0.05
AST	0.21 *	0.44 ***	−0.17	−0.53 ***	0.08	−0.05	−0.14	−0.21 *
Calcium	−0.08	−0.33 ***	−0.03	0.37 ***	−0.09	−0.16	−0.14	0.14

Levels of significance: * *p* < 0.05; ** *p* < 0.01; *** *p* < 0.001. Abbreviations: L*, lightness; a*, redness; b*, yellowness; CL, cooking loss; WBS, Warner–Bratzler shear force; AST, aspartate aminotransferase.

**Table 4:** Correlation coefficients between the serum biochemical parameters and sensory quality traits of broilers.

	IT	RB	APR	OT	J	FI	OFI	OA
Blood glucose	−0.05	−0.05	−0.06	−0.07	−0.01	−0.04	−0.10	−0.04
Serotonin	0.07	0.08	0.17	0.07	0.08	0.18	−0.28 **	−0.12
AST	0.19	0.23 *	0.33 ***	0.20 *	0.27 **	0.26 **	−0.32 ***	−0.25 **
Calcium	−0.07	−0.11	−0.20 *	−0.09	−0.16	−0.17	−0.17	−0.14

Levels of significance: * *p* < 0.05; ** *p* < 0.01; *** *p* < 0.001. Abbreviations: IT, initial tenderness; RB, rate of breakdown; APR, amount of perceptible residue; OT, overall tenderness; J, juiciness; FI, flavor intensity; OFI, off-flavor intensity; OA, overall acceptability; AST, aspartate aminotransferase.

## Data Availability

The data presented in this study are available on request from the corresponding author.
